# Adult Nodal Burkitt Lymphoma Forming Nodular Architectures

**DOI:** 10.7759/cureus.19130

**Published:** 2021-10-29

**Authors:** Yutaka Tsutsumi, Soshi Yanagita, Kouichi Ohshima, Mitsuhiro Tachibana

**Affiliations:** 1 Diagnostic Pathology Clinic, Pathos Tsutsumi, Inazawa, JPN; 2 Department of Diagnostic Pathology, Shimada City General Medical Center, Shimada, JPN; 3 Department of Hematology, Shimada City General Medical Center, Shimada, JPN; 4 Department of Pathology, Kurume University School of Medicine, Kurume, JPN

**Keywords:** nodular architectures, nodal burkitt lymphoma, molecular analysis, immunohistochemistry, follicular colonization

## Abstract

In this report, we discuss a case of nodal Burkitt lymphoma seen in a 60-year-old Japanese male patient. Microscopic features of the biopsied 30 mm-sized cervical lymph node revealed nodular architectures with starry sky appearance surrounded by small mantle zone B-lymphocytes. Immunohistochemical and molecular studies demonstrated typical features of sporadic Burkitt lymphoma: the atypical cells were positive for CD20, CD79a, CD10, CD23, HLA-DR, bcl-6, PAX5, c-myc, and cytoplasmic IgM, but negative for CD3, CD5, CD15, CD30, CD34, TdT, bcl-2, and MUM1. The mantle zone B-cells were clearly positive for bcl-2 and IgD. *In situ*hybridization (ISH) analysis for immunoglobulin light chains showed kappa-type monoclonality. A few nuclei were labeled for Epstein-Barr virus-encoded small nuclear RNA (EBER). Ki-67 labeling index was nearly 100%. Within the nodule, CD21, CD23, and CD35-positive follicular dendritic cells were scattered with a small number of CD3/CD5-positive small T-lymphocytes, indicating that the nodular architecture represented follicular colonization of Burkitt lymphoma cells. Karyotypic analysis revealed t(8;14)(q24;q32), and IGH-MYC fluorescence *in situ* hybridization (FISH) demonstrated IGH-MYC fusion signals. The presentation of follicular colonization was quite unique in Burkitt lymphoma in the present case. Differential diagnosis is also discussed.

## Introduction

Progressive transformation of germinal centers (PTGC) is a benign lymph nodal disorder of unknown etiology associated with follicular hyperplasia, commonly involving cervical lymph nodes [1]. It occurs predominantly in children and young patients but can affect people of all ages. Male predominance is evident [2]. PTGC is histologically characterized by the presence of large, expansile nodules randomly distributed in a background of hyperplastic follicles. It should be distinguished from nodular lymphocyte-predominant Hodgkin's lymphoma and from follicular lymphoma floral variant [3].

We present a case of nodal Burkitt lymphoma seen in an immunocompetent 60-year-old male patient. It is well known that Burkitt lymphoma derives from germinal center B-lymphocytes [4-6]. In the present case, a unique formation of nodular architectures resembling PTGC was quite characteristic. The nodularity may represent follicular colonization (or an early stage of development) of Burkitt lymphoma. Immunohistochemical analysis, *in situ* hybridization (ISH), and molecular analyses confirmed the diagnosis of Burkitt lymphoma.

Burkitt lymphoma, a high-grade B-cell malignancy commonly associated with an extranodal occurrence, diffuse growth without any nodularity, and central nervous system involvement, is classified into three types: endemic (African), sporadic (non-African), and immunodeficiency (AIDS)-associated [4-6]. Burkitt lymphoma is cytologically marked by monomorphous medium-sized malignant cells having one to several distinct nucleoli and basophilic cytoplasm with characteristic fine vacuoles.

Sporadic type Burkitt lymphoma is predominantly seen in children and young adults with a male-to-female ratio of around 2:3 [4-6]. It consists of 40-50% of childhood lymphoma and 1-2% of adult lymphoma and mainly involves extranodal sites such as the ileocecal region, ovary, kidney, and breast. Lymph node involvement is relatively common in adults. Leukemic manifestation as acute lymphoblastic leukemia (L3) is infrequently encountered. The prognosis is particularly poor in adult patients aged 40 years or above.

The unique features of nodal Burkitt lymphoma of sporadic type in the present adult case are the formation of nodular architectures resembling germinal centers. To the best of our knowledge, there is no clear description of such nodularity (follicular colonization) in Burkitt lymphoma in the literature.

## Case presentation

**Figure 1:**
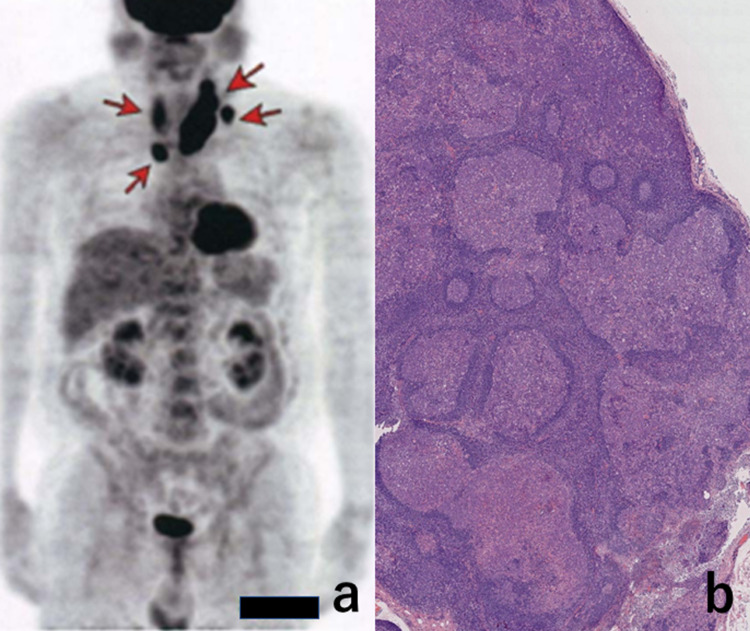
PET/CT scan image (a) and a low-powered microscopic view of biopsied 30 mm-sized cervical lymph node (b: H&E stain) As indicated by arrows, bilateral cervical and upper mediastinal lymph nodes were swollen (a). Irregular-shaped and fused nodular architectures with distinct mantle zone formation are microscopically evident (b) PET: positron emission tomography; CT: computed tomography

A 60-year-old Japanese man experienced swelling of the left-sided neck in July 2020. He visited Shimada General Medical Center, Shimada, Shizuoka, Japan, and malignant lymphoma was suspected following fine-needle aspiration cytology from the enlarged, non-tender cervical lymph node. There were no night sweats, fever, and weight loss. The laboratory data were as follows - white blood cells: 8,800 $\mu$L, hemoglobin: 14.3 g/dL, platelets: 29,7000 $\mu$L, total bilirubin: 0.79 mg/dL, aspartate aminotransferase (AST): 21 IU/L, alanine aminotransferase (ALT): 22 IU/L, lactate dehydrogenase (LDH): 165 IU/L, C-reactive protein: 0.97 mg/dL (standardized value: <0.3), and soluble interleukin-2 receptor: 536 U/mL (standardized value: 190-650). His past medical history revealed hypertension, hyperuricemia, diabetes mellitus, and cataract. No immunodeficiency status was recorded. The patient had smoked 40 cigarettes per day for eight years since the age of 18 years but had stopped thereafter. He infrequently drank alcohol. PET/CT images disclosed multifocal nodal swelling up to 40 mm in size in the bilateral neck regions and upper mediastinum (Figure 1a).

A left cervical lymph node measuring 30 x 20 x 20 mm was excisionally biopsied. Histological features resembled PTGC, but the final diagnosis was nodal Burkitt lymphoma (see below). Bone marrow aspiration disclosed the involvement of atypical lymphoid cells, indicating systemic dissemination of the lymphoma cells (stage IV).

After the cannulation of a central venous catheter, chemotherapy (CODOX-M: cyclophosphamide, vincristine, doxorubicin, and methotrexate) was started in September 2020. Intrathecal injections of methotrexate were added to the regimen. Because of a good response, rituximab (humanized monoclonal antibody against CD20) was not added to the protocol. The second IVAC (ifosfamide, etoposide, and cytarabine) was started in November 2020. During chemotherapy (CODOX-M/IVAC), in two cycles, a herpes zoster virus infection on the left eyelid complicated the procedure. The nodal lesions were retracted, and bone marrow suppression and liver and renal dysfunctions were well controlled. The patient is currently being followed up in an outpatient clinic (October 2021).

Microscopic features of the biopsied lymph node

**Figure 2:**
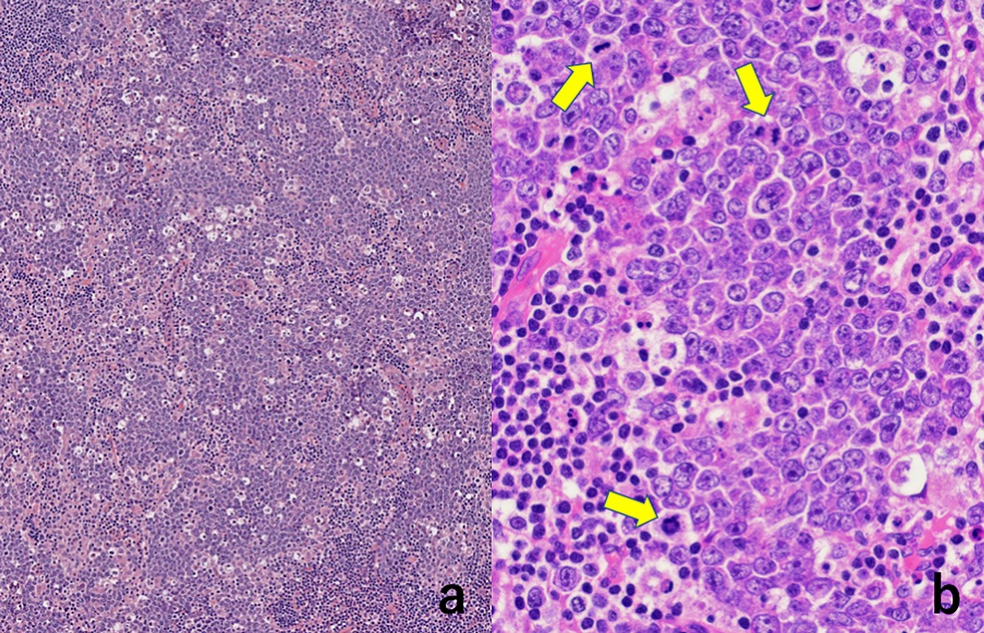
Microscopic appearance of the cervical lymph node (H&E stain) In the nodular architecture, the existence of tingible body macrophages gives a picture of starry sky appearance (a). In a high-powered view (b), the medium-sized monomorphous growing cells possess basophilic cytoplasm and round nuclei with prominent nucleoli. Mitotic activity is easily observed (arrows). Tingible body macrophages phagocytize apoptotic bodies

In the hematoxylin and eosin (H&E) staining, the nodal parenchyma was replaced by irregular-shaped and fused germinal center-like nodal architectures, which were consistently surrounded by mantle zone small lymphocytes (Figure 1b). Tingible body macrophages (showing starry sky appearance) and mitotic pictures were easily noted within the nodules (Figure 2).

**Figure 3:**
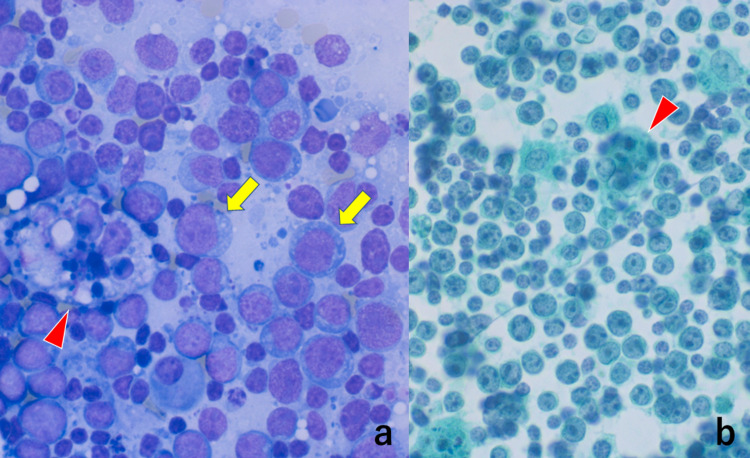
Stamp cytology features of the biopsied lymph node (a: Giemsa, b: Papanicolaou) The medium-sized atypical lymphocytes possess round nuclei with prominent nucleoli. The scanty cytoplasm is basophilic. Fat vacuoles are observed in Giemsa-stained preparation (arrows). Macrophages phagocytizing apoptotic bodies are scattered (arrowheads)

Our original histological diagnosis based on such H&E-stained features was PTGC. Because of the multicentric involvement and high standardized uptake value in the PET/CT scan, malignant lymphoma was strongly suspected clinically. Monomorphous growth of medium-sized atypical lymphoid cells is unusual in PTGC. Stamp cytological preparations revealed that medium-sized atypical lymphoid cells possessed scanty basophilic cytoplasm with fine vacuolation, and macrophages phagocytizing apoptotic bodies were noted (Figure 3).

Immunohistochemical and molecular studies were performed, as follows.

Immunohistochemical and molecular studies

**Figure 4:**
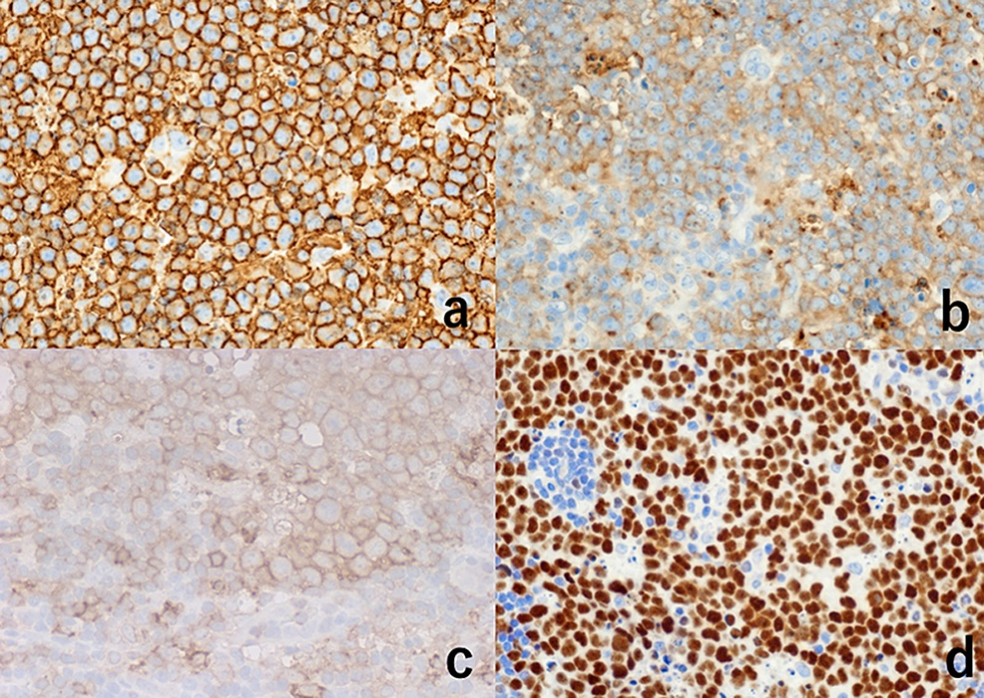
Immunohistochemical features I The proliferating lymphoid cells in the nodule are diffusely immunoreactive for CD20 (a), CD10 (b), HLA-DR (c), and bcl-6 (d)

The monomorphous atypical cells in the nodules were diffusely immunoreactive for CD20, CD79a, CD10, HLA-DR, bcl-6, and PAX5 (Figure 4).

**Figure 5:**
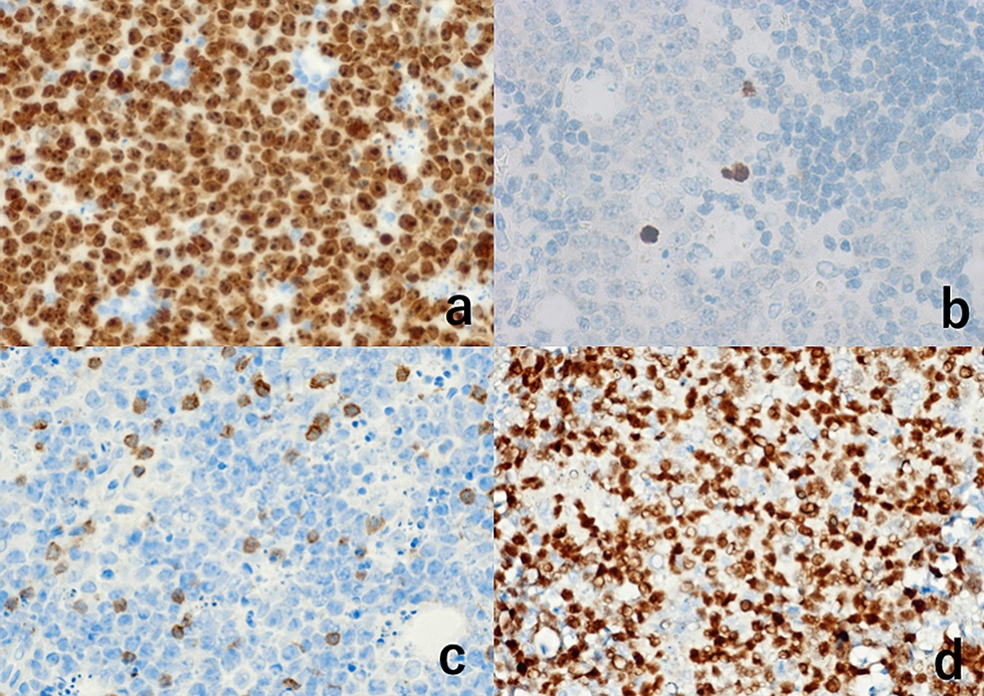
Immunohistochemical and ISH features II (a: Ki-67, b: EBER, c: CD3, d: c-myc) Ki-67 labeling index is nearly 100% in the proliferating cells (a). A few nuclei are labeled with EBER (b). A small number of CD3-positive reactive T-lymphocytes are scattered within the nodule (c). The nuclei are strongly immunoreactive for c-myc oncoprotein (d) ISH: *in situ* hybridization; EBER: Epstein-Barr virus-encoded small RNA

The lymphoma cells were negative for terminal deoxynucleotidyl transferase (TdT), bcl-2, MUM1, CD3, CD5, CD15, CD30, and CD34. Ki-67 labeling index was nearly 100%. By ISH for Epstein-Barr virus-encoded small nuclear RNA (EBER), positive nuclear signals were observed in a few nuclei of the atypical cells in the nodule (Figure 5).

**Figure 6:**
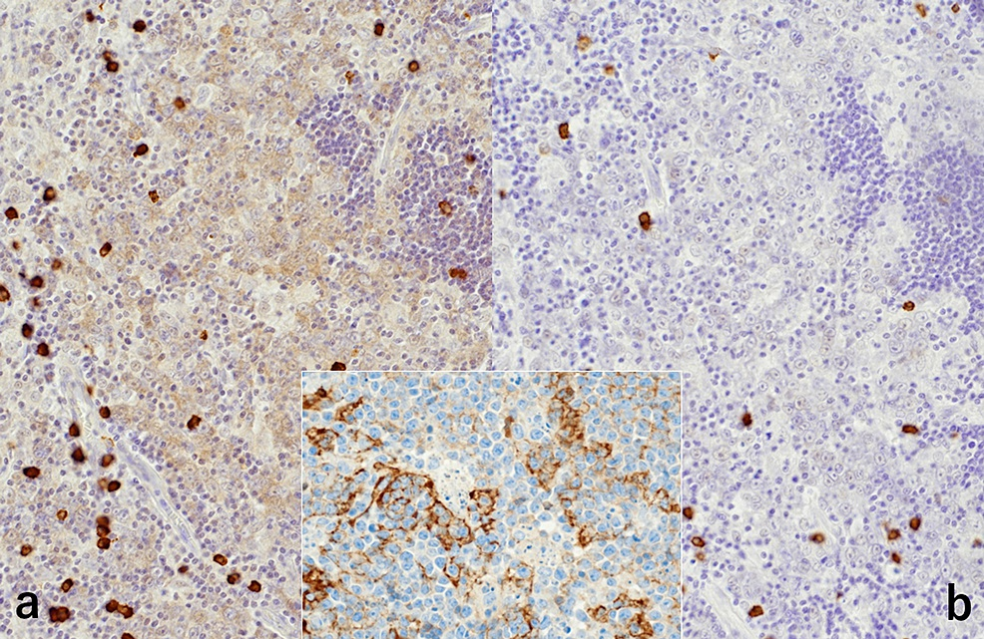
Illustration of light chain restriction: ISH for kappa chain (a) and lambda chain (b). Inset: immunostaining for IgM The cytoplasm of the nodular proliferating cells is faintly positive for kappa chain (a) but negative for lambda chain (b), indicating ISH-mediated kappa-chain monoclonality. The cytoplasmic IgM expression is identified immunohistochemically (inset) ISH: *in situ* hybridization; IgM: immunoglobulin M

Cytoplasmic IgM was demonstrated in the lymphoma cells. ISH study for immunoglobulin light chains showed kappa-type monoclonality (Figure 6).

**Figure 7:**
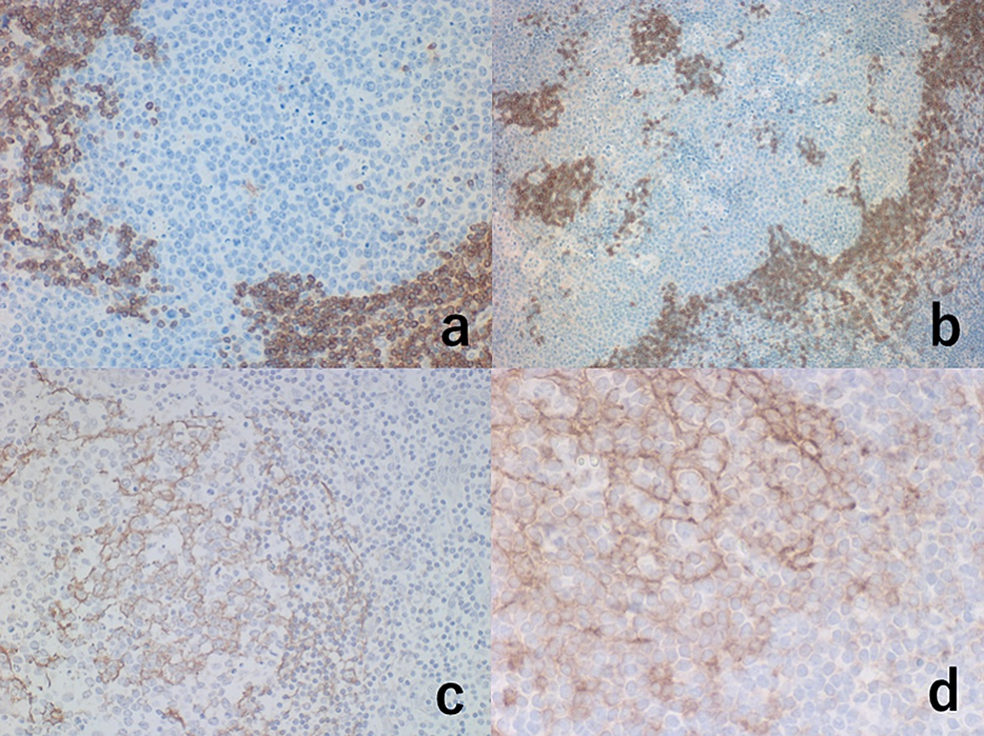
Immunohistochemical features III: the association of mantle zone cells and follicular dendritic cells (a: bcl-2, b: IgD, c: CD21, d: CD23) The formation of mantle zones is evident by immunostaining for bcl-2 (a) and IgD (b). Some mantle cells are entrapped within the growing nodule. The lymphoma cells are negative for bcl-2 and IgD. Within the nodule, follicular dendritic cells immunoreactive for CD21 (c) and CD23 (d) are distributed. The proliferating cells weakly express CD23 (d)

CD3/CD5-positive reactive small T-lymphocytes were scattered in the nodule (Figure 5c). Follicular dendritic cells immunoreactive for CD21, CD23, and less consistently, CD35 were scattered within the nodular architecture, indicating that the nodules represent germinal centers. CD23 was weakly expressed on the lymphoma cells. The mantle zone small lymphocytes were clearly labeled for bcl-2 and IgD (Figure 7).

Some mantle cells were entrapped within the growing nodule.

**Figure 8:**
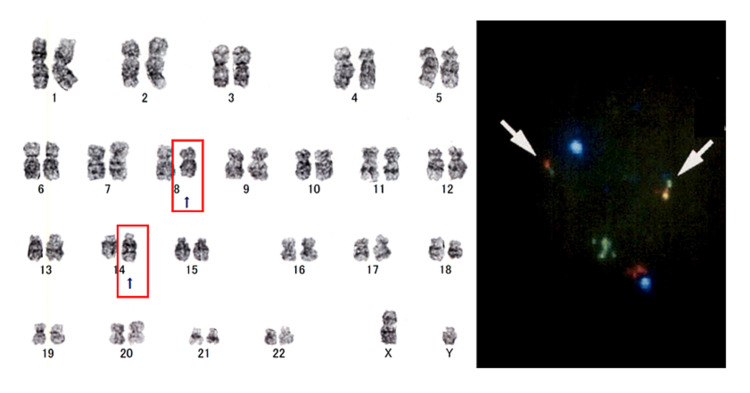
Karyotypic analysis (left) and IGH-MYC FISH (right) The karyotypic analysis demonstrates t(8;14)(q24;q32), as indicated with red boxes. IGH-MYC FISH (IGH signals in green and MYC signals in red) illustrates IGH-MYC fusion signals (arrows) in 14% of cells evaluated FISH: fluorescence in situ hybridization

Karyotypic analysis revealed a translocation t(8;14)(q24;q32), and IGH-MYC fluorescence *in situ* hybridization (FISH) demonstrated IGH-MYC fusion signals in 14% of cells (Figure 8).

c-myc protein was strongly immunoreactive in the nuclei of the lymphoma cells (Figure 5d).

All these features were typical and diagnostic of Burkitt lymphoma.

## Discussion

We described a case of sporadic Burkitt lymphoma occurring in the cervical lymph node of a 60-year-old Japanese man. Immunohistochemical and molecular features were typical of Burkitt lymphoma with kappa-type monoclonality, as described above. The formation of germinal center-like nodular architectures surrounded by mantle zone small B-lymphocytes was quite characteristic in the present case. Because of the presence of follicular dendritic cells in the nodule, we can regard it as follicular colonization by the Burkitt lymphoma cells. EBER was focally positive in the nuclei. Regarding EBV etiology, EBER is nearly 100% positive in endemic types, infrequent (less than 30%) in sporadic types, and 25-40% in immunodeficiency-associated types [4-6].

It is well known that Burkitt lymphoma cells share features of germinal center B-lymphocytes, but they commonly involve the nodal and extranodal lesions with a massive and diffuse fashion [4-6]. To the best of our knowledge, nodal involvement of Burkitt lymphoma with nodular architectures has not clearly been described in the literature. Gonzalez-Farre et al. [7] described such a nodularity in a case of Burkitt-like lymphoma of germinal center origin. It is noteworthy that follicular lymphoma may transform to Burkitt or Burkitt-like lymphoma [8-10]. In the present case, there was no evidence of pre-existing follicular lymphoma. The involvement of germinal centers (follicular colonization) by Burkitt lymphoma should thus be regarded as an early-stage involvement, we believe.

Follicular colonization has been described in mucosa-associated lymphoid tissue (MALT) or marginal zone lymphoma [11,12]. Infrequently, diffuse large B-cell lymphoma of the tonsil shows follicular colonization, and hence differential diagnosis from follicular lymphoma is needed [13,14]. It is noteworthy that Burkitt lymphoma may show follicular colonization.

At first, we made a diagnostic misjudgment as PTGC, because of the distinct nodular growth pattern and negativity of bcl-2 expression. PTGC is a benign and reactive lymphadenopathy mainly encountered in the cervical lymph node [1-3]. It predominantly occurs in male patients, and two-thirds of cases accompany a solitary lesion. Chronic inflammation in the head and neck region and autoimmune disorders may be associated with it. Enlarged germinal centers, two to four times larger than normal, are often infiltrated by mantle zone small lymphocytes. Differential diagnosis of PTGC includes nodular lymphocyte-dominant Hodgkin’s lymphoma and the floral variant of follicular lymphoma [3]. We propose that Burkitt lymphoma forming nodular architectures should be added to the list of differential diagnoses for PTGC.

## Conclusions

We discussed a rare case of adult nodal Burkitt lymphoma forming nodular architectures or follicular colonization. For appropriate histopathological diagnosis. we should be aware of such a unique variation in the microscopic features of Burkitt lymphoma, a high-grade B-cell lymphoma of germinal center cell origin. PTGC should be prominently listed as part of the differential diagnosis.
